# LIGHT enhanced bispecific antibody armed T-cells to treat immunotherapy resistant colon cancer

**DOI:** 10.1038/s41388-022-02209-w

**Published:** 2022-02-17

**Authors:** Guilin Qiao, Lyonell B. Kone, Evan H. Phillips, Steve Seung-Young Lee, Grace E. Brown, Salman R. Khetani, Archana Thakur, Lawrence G. Lum, Bellur S. Prabhakar, Ajay V. Maker

**Affiliations:** 1 Department of Surgery, Division of Surgical Oncology, University of California San Francisco, San Francisco, CA 94143 USA; 2 Department of Surgery, Division of Surgical Oncology, University of Illinois at Chicago, College of Medicine, Chicago, IL 60612 USA; 3 Department of Pharmaceutical Sciences, College of Pharmacy, University of Illinois at Chicago, Chicago, 60612 USA; 4 Department of Bioengineering, University of Illinois at Chicago, Chicago, IL 60607 USA; 5 Division of Hematology/Oncology, Department of Medicine, University of Virginia Cancer Center, Charlottesville, VA USA; 6 Department of Microbiology & Immunology, University of Illinois at Chicago, College of Medicine, Chicago, IL 60612 USA

**Keywords:** Gastrointestinal cancer, Tumour immunology

## Abstract

**Abstract:**

Increased tumor infiltrating lymphocytes (TIL) are associated with improved patient responses to immunotherapy. As a result, there is interest in enhancing lymphocyte trafficking particularly to colon cancers since the majority are checkpoint blockade-resistant and microsatellite stable. Here, we demonstrate that activated T-cells (ATC) armed with anti-CD3 x anti-EGFR bispecific antibody increases TIL and mediate anti-tumor cytotoxicity while decreasing tumor cell viability. Furthermore, treatment induces endogenous anti-tumor immunity that resisted tumor rechallenge and increased memory T-cell subsets in the tumor. When combined with targeted tumor expression of the tumor necrosis factor superfamily member LIGHT, activated T-cell proliferation and infiltration were further enhanced, and human colorectal tumor regressions were observed. Our data indicate that tumor-targeted armed bispecific antibody increases TIL trafficking and is a potentially potent strategy that can be paired with combination immunotherapy to battle microsatellite stable colon cancer.

**Significance:**

Enhancing trafficking of tumor infiltrating lymphocytes (TILs) to solid tumors has been shown to improve outcomes. Unfortunately, few strategies have been successful in the clinical setting for solid tumors, particularly for “cold” microsatellite stable colon cancers. In order to address this gap in knowledge, this study combined TNFSF14/LIGHT immunomodulation with a bispecific antibody armed with activated T-cells targeted to the tumor. This unique T-cell trafficking strategy successfully generated anti-tumor immunity in a microsatellite stable colon cancer model, stimulated T-cell infiltration, and holds promise as a combination immunotherapy for treating advanced and metastatic colorectal cancer.

## Introduction

The estimated 5-year survival of metastatic colorectal cancer (CRC) is a dismal 13%, resulting in it being the second leading cause of cancer-related death worldwide [1, 2]. Perhaps what is most alarming is that treatment strategies have remained largely unchanged over the decades [3] while CRC incidence has rapidly increased in adults 20–55 years old [4]. Therefore, the need for novel treatment paradigms cannot be overemphasized.

Given success in other tumor histologies, there is great enthusiasm to apply new immunotherapeutic strategies; however, durable clinical responses in CRC have been limited to a small minority of patients and only in microsatellite instable disease, which comprises <5% of all colon cancers [5, 6]. What has been established by recent trials is that patients with high numbers of tumor infiltrating lymphocytes (TIL) in the tumor microenvironment (TME) experience increased sensitivity to checkpoint blockade [7, 8] and chemotherapy [9]. Patient survival in CRC is determined by the number and location of TIL trafficking to primary and metastatic CRC tumors [7, 10–17], and we further demonstrated that CRC tumor regressions only occur when T-cells infiltrate the tumors [18, 19]. When the number of TIL were increased, durable anti-tumor regressions with immune-recall responses were possible [18, 20]. Thus, novel strategies that increase TIL trafficking hold great potential to improve outcomes in advanced CRC.

In order to traffic lymphocytes to the TME, a tumor specific antigen is needed. Though tumor reactive T-cells can be found occasionally and expanded from CRC, these are patient and tumor specific, which limits their widespread use. Epidermal growth factor (EGFR) is a validated therapeutic target overexpressed in 65–75% of CRC tumors and ~85% of metastatic CRC tumors [21, 22]. Thus, EGFR may be utilized to draw cytotoxic T-cells into the TME.

Bispecific antibodies (BsAb) can be engineered to promote such trafficking by targeting both an immune cell and a cancer-specific antigen [23]. In this study, a chemically heteroconjugated anti-CD3 antibody is fused to the anti-EGFR antibody and used to produce BsAb armed activated T-cells (BATs). Arming T-cells with BsAb converts these activated T-cells (ATC) into specific T-cell clones without the requirement of tumor-antigen presentation. We have demonstrated the ability to engineer these BsAb, to arm them ex vivo with ATC, and previously established clinical safety with this approach [24–33]. Unlike the majority of other BsAb, this ex vivo arming approach significantly decreases immunotherapy-related side effects, making it an ideal candidate for clinical therapy [34].

We then wished to identify mechanisms to support TIL trafficked by the BsAb. LIGHT (TNFSF14) is an immunostimulatory cytokine required for the activation of CD8 T-cells that can augment the anti-tumor immune response [35, 36]. We previously demonstrated that enhancing tumor necrosis factor superfamily member LIGHT in the TME supported T-cell proliferation, activation, and infiltration in CRC [18]. Increased LIGHT expression decreased CRC recurrence by enabling the host to mount, and the tumor to support, lymphocyte proliferation and cytotoxicity [16]. Hence, we hypothesized that combining BATs with LIGHT would further enhance T-cell trafficking, cytotoxic activity, and proliferation in colorectal cancer. Our study shows that BAT immunotherapy increases TIL, mediates anti-tumor cytotoxicity, and induces endogenous anti-tumor immunity, which, when combined with targeted treatment with tumor necrosis factor superfamily member LIGHT, further enhances anti-tumor immune responses in this otherwise “cold” tumor histology.

## Results

### Establishment of EGFR and LIGHT expressing murine and human microsatellite-stable colorectal cancers

**Fig. 1:**
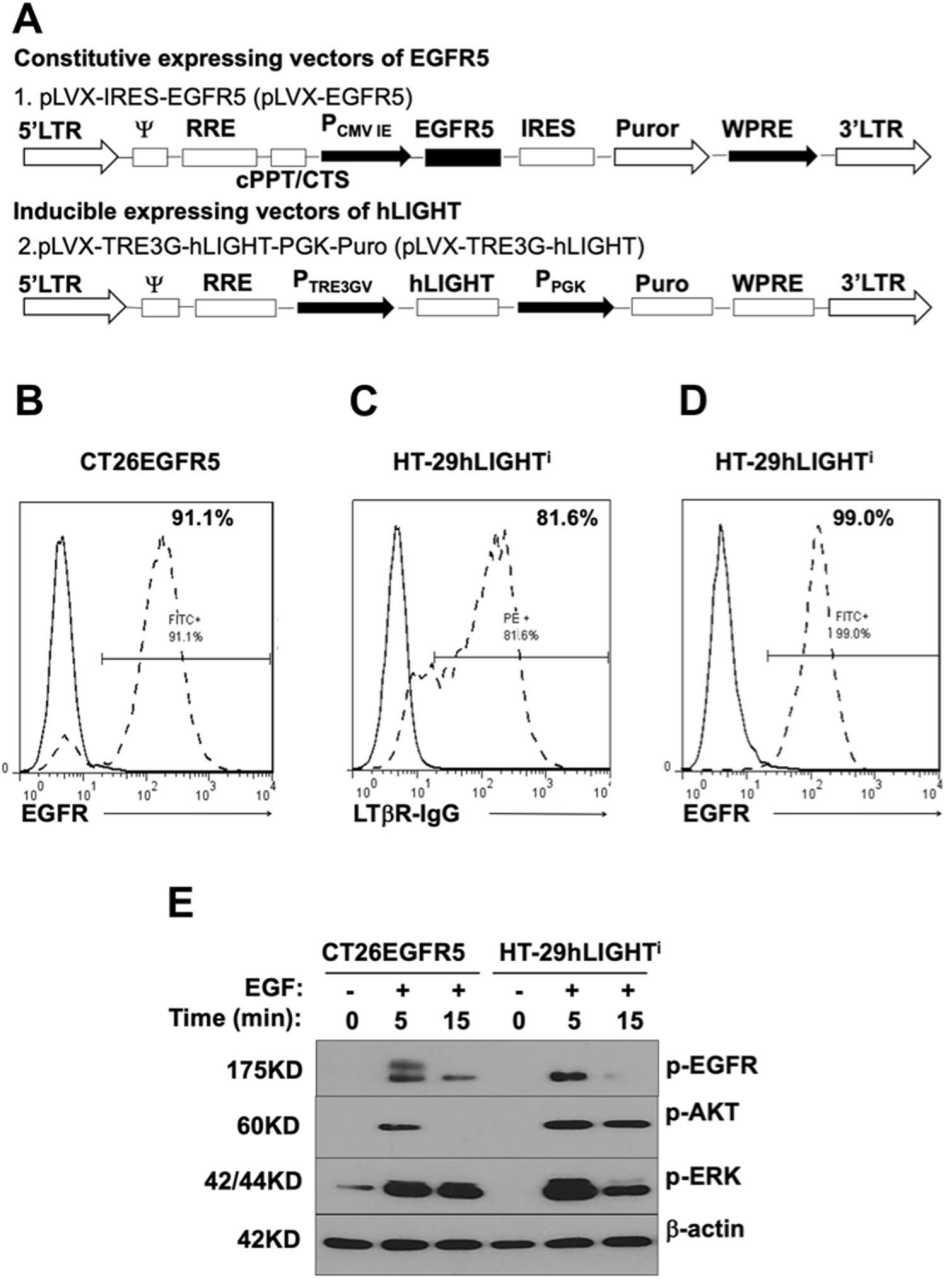
Establishment of EGFR and LIGHT expressing murine and human microsatellite-stable colorectal cancers. **A** EGFR5, mouse EGFR with site mutations enabling Cetuximab binding, was inserted into XhoI sites of the lentiviral expression vector pLVX-IRES-Puro. The human LIGHT gene was inserted into EcoRI and BamHI sites of the inducible lentiviral expression vector pLVX-TRE3G. **B** Stable constitutive expressing EGFR5 murine colon cancer cell line CT26EGFR5 was created in which more than 90% of CT26EGFR5 cells express EGFR5. **C** HT-29LIGHT^i^ human colon cancer cell line was created which inducibly expresses human LIGHT. **D** 99% of HT-29LIGHT^i^ cells express EGFR. **E** CT26EGFR5 and HT-29hLIGHT^i^ cells were stimulated with EGF at 100 ng/ml for 5 and 15 min after serum starvation, and cell lysates resolved on sodium dodecyl sulfate-polyacrylamide gel electrophoresis. The membrane was blotted with phospho-anti-EGFR, phospho-anti-Akt, phospho-anti-ERK, and anti-βactin antibodies.

Xenograft models have been used to evaluate human antibody-based treatments; however, evaluation of immune-mediated regulation of anti-EGFR antibody (Cetuximab) effects are limited since human EGFR-dependent tumors cannot grow in immune-competent wild type mice and there is no mouse tumor cell line that responds to cetuximab. To overcome this obstacle, and, more importantly, enable evaluation of tumor immunity in an intact immunocompetent syngeneic system with normal immune elements, a CT26 murine microsatellite stable (MSS) colon cancer was established with EGFR5, a receptor to which both murine and human anti-EGFR can functionally interact (Fig. 1A). This murine CRC therefore reflects the majority of human CRC and can be evaluated in a fully syngeneic Balb/C system. To further evaluate translational applicability and ability to interact with human T-cells, MSS endogenously EGFR^+^ HT29 human CRC were utilized, into which inducible human LIGHT expression was established (Fig. 1A).

EGFR and LIGHT surface expression and binding to Cetuximab and LTbR (the cognate LIGHT receptor) were confirmed by flow cytometry (Fig. 1B–D). Though downstream signaling is not necessary for the intended function of the BsAb strategy to traffic T-cells into the TME, it is pertinent to the impact of anti-EGFR treatment alone, therefore, downstream pathways were evaluated. EGFR signaling remained intact in CT26EGFR and HT-29hLIGHT^i^ cells with increased phospho-AKT and ERK after EGF stimulation (Fig. 1E). Therefore, as is seen in our CRC patients, the anti-tumor effects of anti-EGFR therapy alone could be expected, and differences in T-cell trafficking and immunity could be directly compared to the BsAb strategy.

### Engineered BsAb binds simultaneously to both CD3 T-cells and colon cancer cells

**Fig. 2:**
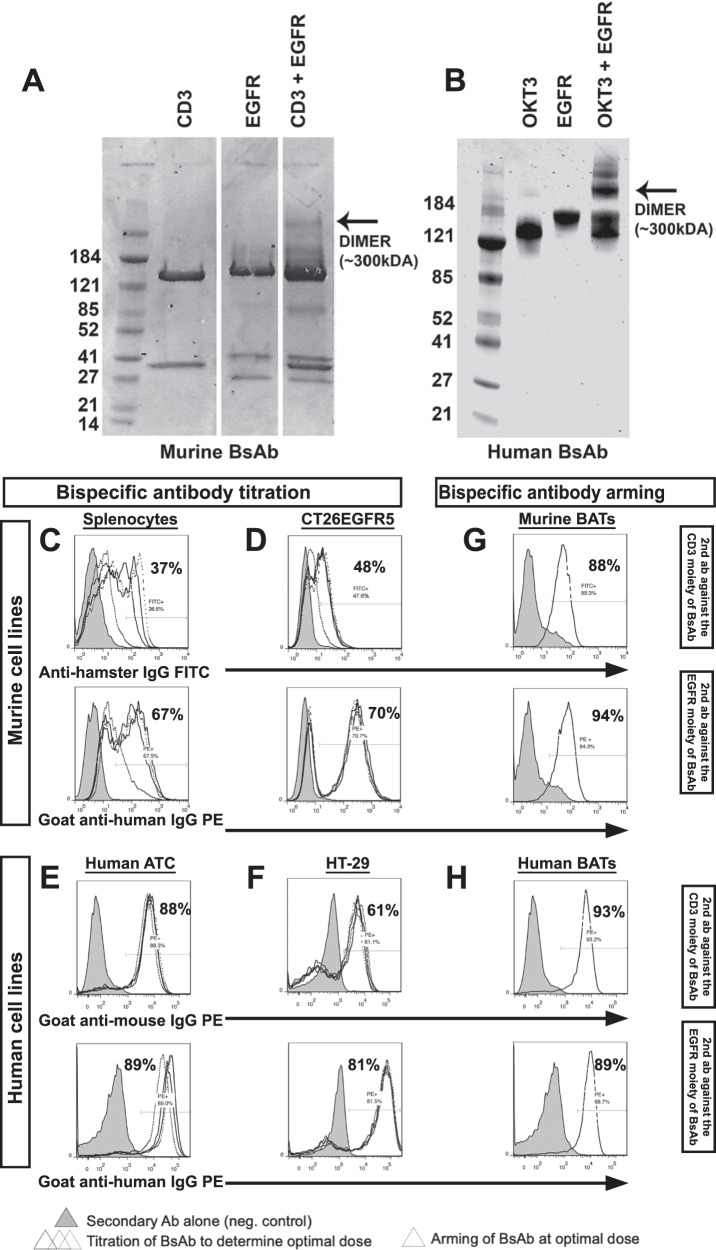
Engineered bispecific antibody binds simultaneously to both CD3 T-cells and colon cancer cells. **A**, **B** Murine and human anti-CD3 and anti-EGFR (Cetuximab) were heteroconjugated. The products were resolved by SDS-nonreducing polyacrylamide gel electrophoresis and bispecific antibody (BsAb) dimers detected by Coomassie blue staining (arrow). **C**–**F** represent the crosslinking and binding efficiency of murine and human BsAb to anti-CD3 and anti-EGFR. Different dosages (25, 50, 100, 200, 500, 1000 ng) of BsAb were added to 1 × 10^6^ murine splenocytes, CT26EGFR5 cells, human activated T cells (ATC) and HT-29 cells. The bispecific antibody bound to surface EGFR or CD3 was detected with directly conjugated anti-hamster IgG-FITC (murine anti-CD3), goat anti-human IgG –PE (murine anti-EGFR), anti-mouse IgG-PE (human anti-CD3) and goat anti-human IgG –PE (human anti-EGFR). **G**, **H** At the optimal concentration of 200 ng/1 × 10^6^ cells for murine BsAb and 50 ng/1 × 10^6^ cells for human BsAb, bispecific antibody is efficiently armed with murine and human ATC (BATs).

Anti-CD3 x anti-EGFR BsAb was produced using heteroconjugation of OKT3/anti-CD3 and Cetuximab [29, 37]. Gel electrophoresis confirmed the presence of heteroconjugated dimers (Fig. 2A, B) and optimal arming concentrations for T-cells were established by evaluating anti-CD3 antibody binding with secondary antibodies to EGFR, and anti-EGFR antibody binding with secondary antibodies to CD3 (Fig. 2C–F) as we have previously described [29, 37]. Similarly, murine ATC armed with anti-CD3 x anti-EGFR BsAb were 88% CD3 moiety positive and 94% EGFR-moiety positive (Fig. 2G). Armed human ATC were 93% CD3 moiety positive and 89% EGFR-moiety positive (Fig. 2H). Therefore, both the murine and human BsAb bound simultaneously to CD3 on ATC and EGFR on tumor cells, and BsAb armed ATC were capable of binding to murine CT26 and human HT29 EGFR expressing CRC tumors. To further demonstrate that BsAb bound simultaneously to both CD3 T-cells and colon cancer cells, CD3+ T cells were labeled with cell tracker violet, and murine and human cancer cells were labeled with far red. Co-incubation with BsAb in the presence or absence of excess amounts of anti-CD3 and anti-EGFR confirmed that BsAb specifically and simultaneously bound CD3+ T cells and EGFR+murine CT26EGFR5 or human HT-29 cells.

### BATs increase T-cell trafficking and cytotoxicity resulting in decreased tumor cell viability in murine colorectal cancer

**Fig. 3:**
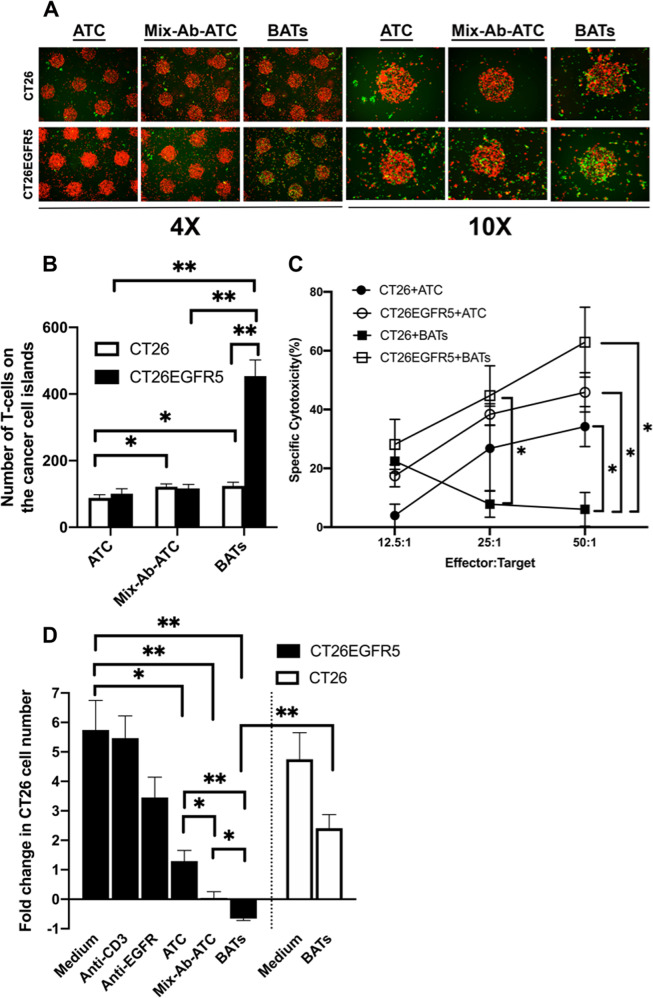
BATs increase T-cell trafficking into murine colon cancer micro-island organoids, mediate increased T-cell cytotoxicity, and decrease tumor cell viability. **A**, **B** CT26 or CT26EGFR cells were stained with far red in micro-island organoids. ATC were armed with BsAb (BATs) at 200 ng/1 × 10^6^ cells/100 µl or a mixture of anti-CD3 (500 µg/1 × 10^6^ cells/100 µl) and anti-EGFR (500 µg/1 × 10^6^ cells/100 µl) (Mix-Ab-ATC) after CFSE labeling. T-cell trafficking was substantially increased in EGFR expressing tumors when treated with BATs. **C** Cytotoxicity mediated by murine ATC or BATs to CT26 or CT26EGFR cells was performed with a non-radioactive calorimetric cytotoxicity assay. **D** Anti-proliferation effects of BATs on CT26 and CT26EGFR were quantified. Cells were harvested in triplicates and quantified 48 h after coculture.

In order to determine the ability of BATs to traffic to CRC cells, CT26 micro-island organoids were bioengineered in vitro. The cancer cells were labeled with far red and seeded in micropatterned collagen type I circular 3-D domains. After just 1 h, there was a significant 4.5-fold increase in TIL in the CT26EGFR islands treated with BATs compared to ATC or ATC plus both anti-T cell and anti-EGFR antibodies (Fig. 3A, B).

Tumor cells were co-cultured with BATs and cytotoxicity determined at effector target ratios (E:T) from 12.5–50:1. In wtCT26, there was minimal T-cell trafficking or binding to tumor cells, resulting in negligible T-cell mediated cytotoxicity (Fig. 3C) or changes in cell viability (Fig. 3D), as would be expected without EGFR expression. However, in CT26EGFR cultures, cytotoxicity was significantly increased from 6% to 63% compared to wtCT26 control (*p* = 0.01) (Fig. 3C). Furthermore, CT26EGFR cells cultured with medium, anti-CD3, anti-EGFR, ATC, and mixed antibody-ATC all proliferated, albeit less when in the presence of ATC. Not all T-cells that traffic to the tumor will necessarily convey cytotoxicity. Though not significantly greater than +ATC alone, cytotoxicity is greatest in the CT26EGFR+BATs group, and only CT26EGFR cells treated with BATs experienced *decreased* viability (−0.65-fold change, *p* = 0.003) (Fig. 3D). The proliferation of cells in the coculture at 48 h confirmed that tumor cell proliferation is not only significantly abrogated, but actually decreases between BAT and ATC treated groups.

### BATs treatment increases intratumor T-cell trafficking in vivo and generates anti-tumor immunity in a syngeneic system

**Fig. 4:**
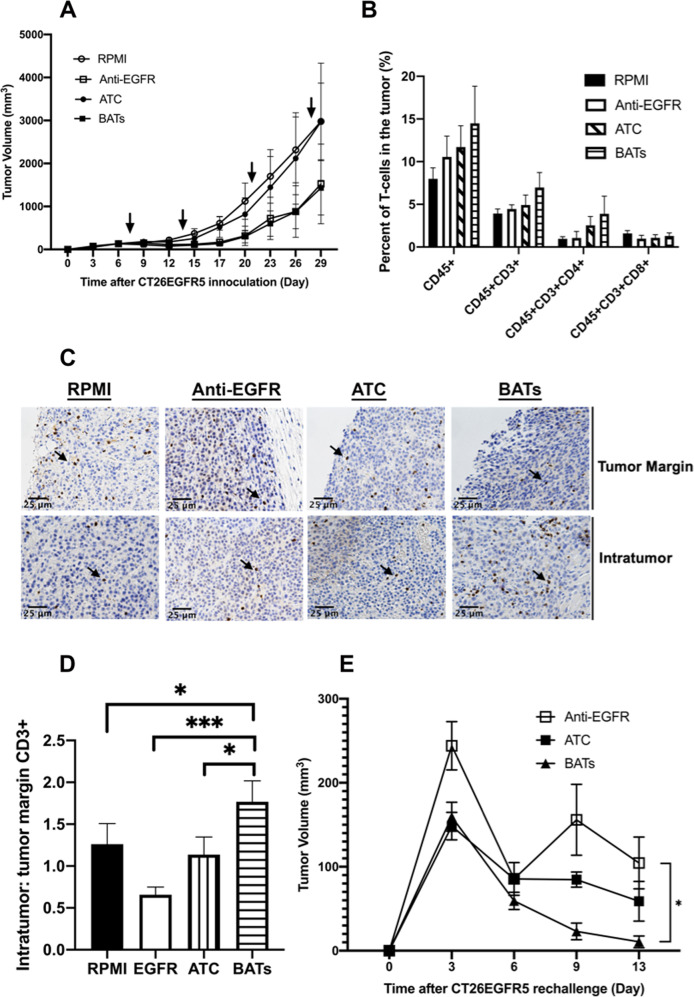
BATs treatment increases intratumor T-cell trafficking in vivo and generates anti-tumor immunity in a syngeneic system. **A** Once CT26EGFR tumors were palpable, animals were randomized and adoptively transferred with systemic administration of 100 µl of RPMI, anti-EGFR, ATC, or BATs (arrows). **B** Single cell tumor suspensions (1 × 10^6^/sample) on day 29 were analyzed with FACS after staining with conjugated antibodies for T cells and T cell subsets. **C** To determine the architectural distribution of tumor infiltrating lymphocytes (TIL), TIL was identified and quantified with immunohistochemistry (arrows demonstrate CD3+ TIL). **D** The greatest number of T-cells and largest ratio of intratumor to tumor margin CD3^+^ T cells were in tumors treated with BATs. **E** 14 days after the last treatment intervention (day 43), mice were challenged with CT26EGFR5 (5 × 10^6^ cells/injection) cells subcutaneously in the left flank. Mice that had been treated with BATs demonstrated complete or near-complete tumor rejection compared to mice treated with Cetuximab monotherapy.

CT26EGFR tumors were established in the flank of immunocompetent syngeneic mice and allowed to grow until palpable. Though treatment effects would be more difficult to demonstrate with a larger tumor burden, the model more accurately reflected how our patients present prior to initiation of treatment. Furthermore, since these animals retained an intact immune system, this model enabled evaluation of the various treatments on anti-tumor immunity. Animals were randomized to four treatment groups and treated weekly with systemic administration of medium, ATC, BATs, or Cetuximab. Tumor size with ATC treatment alone was nearly identical to treatment with media. Similarly, tumor size with Cetuximab treatment, a known cytotoxic chemotherapy, was nearly identical to treatment with BATs (Fig. 4A). Flow cytometry of single cell tumor suspensions to evaluate for TIL revealed a trend towards greater infiltration of CD45+, CD45+ CD3+ and CD4+ cells with BATs compared to all other treatment groups that neared significance (*p* = 0.057) (Fig. 4B). In order to evaluate the ability of BsAb to specifically increase TIL trafficking past the peritumoral milieu and into the center of the tumor, a known prognostic feature of improved survival and response to immunotherapy in CRC, a closer look at the architectural distribution of T-cell trafficking in vivo was necessary. Immunohistochemistry revealed that the proportion of cells reaching the tumor center, as opposed to remaining at the margin, was significantly increased only in animals treated with BATs (Fig. 4C, D).

To determine if the increase in T-cell trafficking with BsAb could generate anti-tumor immunity, animals that had not reached humane endpoints were rechallenged at 6 weeks with tumor inoculation. Only those animals that had been treated with BATs experienced complete or near-complete tumor rejections. It is important to note that though Cetuximab impacted primary tumor growth similarly to BAT treatment, there was no comparable ability to reject tumor rechallenge, i.e., generate anti-tumor immunity (*p* = 0.04) (Fig. 4E).

### BATs increase T-cell trafficking and cytotoxicity resulting in decreased tumor cell viability in wild-type and LIGHT expressing human colorectal cancer

**Fig. 5:**
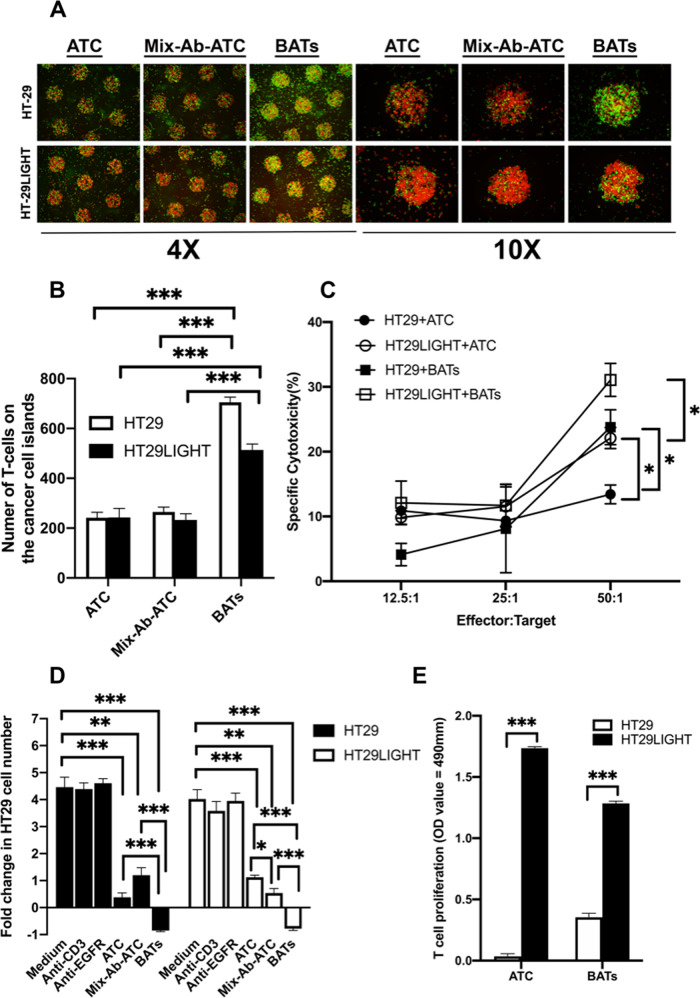
BATs increase T-cell trafficking into colon cancer micro-island organoids, mediate increased T-cell cytotoxicity, and decrease tumor cell viability in wild type and LIGHT expressing human colorectal cancer cells. **A**, **B** HT29hLIGHT^i^ human colon cancer cells were cultured in absence or presence of doxycycline at 1 µg/ml for 24 h used as HT29 and HT29LIGHT cells, respectively, and stained with far red in micro-island organoids. ATC were armed with BsAb (BATs) or a mixture of anti-CD3 and anti-EGFR (Mix-Ab-ATC) after CFSE labeling. T-cell trafficking was substantially increased in tumors when treated with BATs. **C** Cytotoxicity mediated by human ATC or BATs to HT29 or HT29LIGHT cells was determined with a non-radioactive calorimetric cytotoxicity assay. **D** Anti-proliferation effects of BATs on HT29 and HT29LIGHT were quantified. Cells were harvested in triplicates and quantified 48 h after coculture. **E** Human ATC and BATs proliferation were assessed using the MTS method after coculture with irradiated HT-29 or HT-29LIGHT cells for 5 days.

Human CRC micro-island organoids were bioengineered, labeled, and seeded as in the murine experiment. Similarly, there was a significant 2.9-fold increase in BATs in the HT29 islands compared to ATC alone or mixed antibody armed ATC (Fig. 5A, B). Furthermore, akin to the murine results, BATs significantly increased cytotoxicity from 13% to 24% compared to ATC alone (*p* = 0.03), and decreased HT29 cell viability (−0.85, *p* = 0.0001) compared to medium, anti-CD3, anti-EGFR, ATC, and mixed antibody-BsAb treatment (Fig. 5C, D).

After tumor necrosis factor superfamily LIGHT exposure, BATs similarly enhanced T-cell trafficking into tumor micro-island organoids, increased in vitro cytotoxicity, and decreased tumor cell proliferation compared to ATC alone or mixed antibody-ATC (Fig. 5A–D). As ATC are by definition already activated ex vivo with OKT3 and IL2 prior to BsAb arming, and LIGHT functions as an activating cytokine, the findings were as expected. However, the greatest cytotoxicity was present with HT29LIGHT+BATs. Therefore, the ability of LIGHT to support TIL propagation was further evaluated. LIGHT expressing HT29 cells significantly increased T-cell proliferation in both ATC and BATs groups in vitro compared to non-LIGHT expressing cells. (HT-29LIGHT: ATC = 1.74, BATs = 1.28; HT-29: ATC = 0.035, BATs = 0.35; *p* = <0.001) (Fig. 5E).

### BATs treatment increases CD8^+^ T-cell tumor infiltration of human CRC tumors in vivo and generates memory T-cells in a xenograft model

**Fig. 6:**
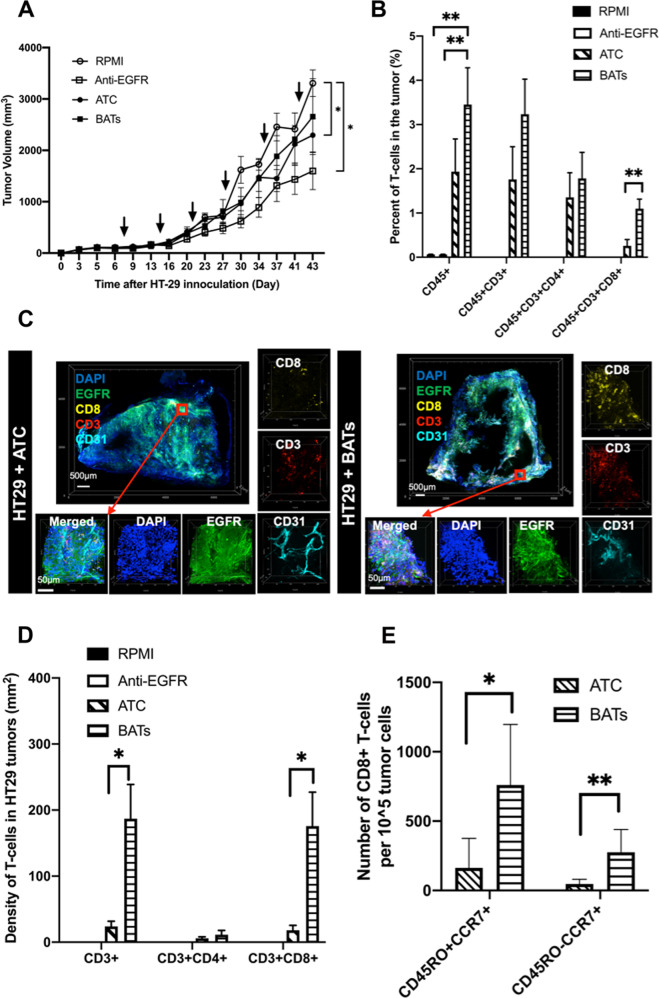
BATs treatment increases CD8+ T-cell tumor infiltration of human CRC tumors in vivo and generates memory T-cells in a xenograft model. **A** HT-29 tumors were established in the right flank of NSG mice. When the palpable tumors reached 150 mm^3^, the mice were randomized. Groups were treated systemically with RPMI, anti-EGFR, ATC, or BATs. **B** Single cell tumor suspensions were analyzed by FACS for T-cell quantification. **C** Transparent tissue tomography revealed markedly increased infiltration of CD3^+^ CD8^+^ T-cells co-localizing with EGFR tumor expressing tissue in BATs treated tumors compared to ATC treated alone (representative image). **D** Tumor infiltrating T cells were stained with human anti-CD3, anti-CD4 and anti-CD8 on tumor sections and quantified. **E** CD8+ tumor infiltrating lymphocytes were further evaluated with FACS for CD45RO and CCR7 expression enabling quantification of memory subsets.

In order to evaluate human CRC cells with endogenous human EGFR expression, HT29 cell tumors were established in immunodeficient mice. Though this model does not allow for evaluation of long-term tumor recall, as the syngeneic murine system enabled, it facilitated evaluation of the impact of BsAb on ATC-specific trafficking and phenotype since there are no circulating T-cells in control or anti-EGFR alone treated animals. Similar to murine CRC in vivo, there was no statistically significant difference in tumor growth of human CRC treated with anti-EGFR compared to BATs (Fig. 6A), as would be expected in an EGFR+ colon cancer with intact downstream signaling. However, the trend toward increased T-cell trafficking with BATs observed in murine tumors was significantly reproduced. The trend of increasing infiltration of BATs compared to ATC groups persisted across all CD45+ subsets evaluated, and it was significant in CD8+ TIL (4.2-fold, *p* = 0.009) (Fig. 6B). This finding was confirmed on 3-D immunohistochemistry where CD8+ TIL were substantially increased at 176 cells/mm^2^ in the BATs group compared to 18 cells/mm^2^ in the ATC alone group (*p* = 0.01) (Fig. 6C, D).

Since the CD8+ T-cell population was specifically increased with BATs treatment compared to ATC alone, further evaluation of the phenotype of these CD8+ TIL was performed. Flow cytometry analysis revealed that the proportion of CD45RO^−^CCR7^+^ stem memory and CD45RO^+^CCR7^+^ central memory cells were significantly increased in the BATs group (Fig. 6E) with a 5-fold increase in stem-cell memory T-cells (*p* = 0.007) and 4-fold increase in central memory T-cells (*p* = 0.013).

### LIGHT imparts HT29 human CRC tumor regressions, and increases both CD4^+^ and CD8^+^ ATC tumor infiltration

Since in vitro evaluation revealed that LIGHT supported proliferation of ATC (Fig. 5), we then aimed to determine if the T-cell response, particularly the CD8+ T-cell response, and anti-tumor immunity could be further enhanced with LIGHT in vivo.

**Fig. 7:**
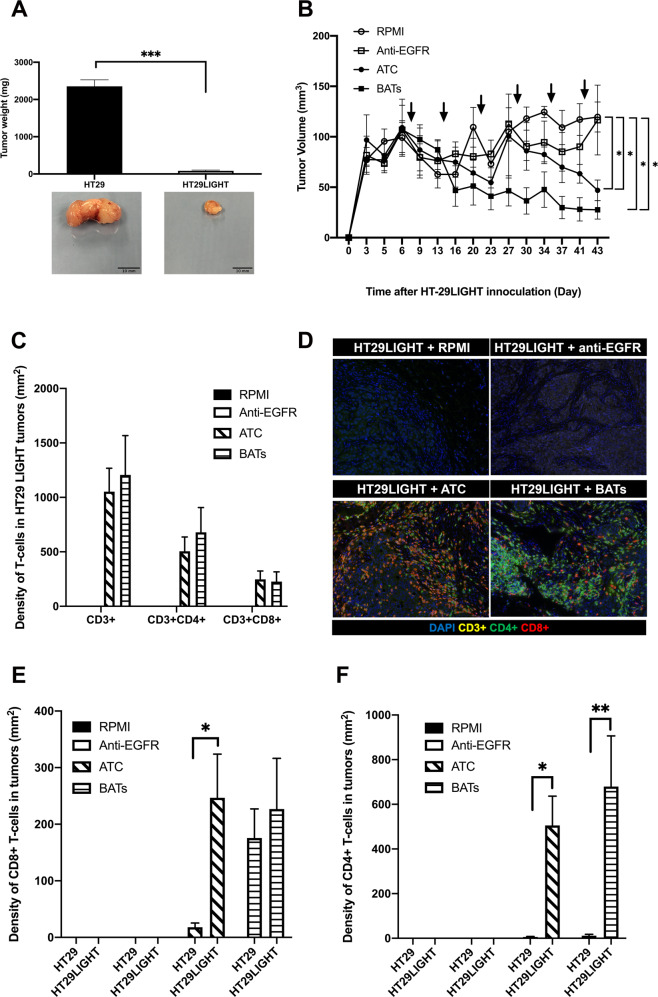
Tumor necrosis factor superfamily LIGHT enhances T-cell proliferation in vitro, imparts HT29 human CRC tumor regressions in vivo, and increases ATC tumor infiltration. **A** HT-29 human CRC tumors were established in the right flank of NSG mice. When the palpable tumors reached 150 mm^3^, LIGHT expression was induced in half the tumors and the mice were randomized. Groups were treated systemically with RPMI, anti-EGFR, ATC, or BATs. Tumor volume and weight were significantly decreased in all groups with LIGHT expression. **B** Tumor growth curves for HT-29LIGHT tumors are depicted with treatment days indicated by arrows. **C**, **D** T-cell density was quantified with fluorescent immunohistochemistry from whole tumor preparations with human anti-CD3, anti-CD4, and CD8. **E**, **F** Density of CD8+ and CD4+ tumor infiltrating lymphocytes after treatment with ATC or BATs is compared between non-LIGHT and LIGHT expressing HT29 CRC tumors.

After HT29 tumors were palpable and reached sufficient size, LIGHT was inducibly expressed. Tumor mass after 43 days was significantly decreased in LIGHT expressing compared to non-LIGHT expressing tumors. (average tumor weight in control animals was 83 vs. 2352 mg, *p* < 0.001) (Fig. 7A). LIGHT expressing tumors all experienced decreased growth rates compared to non-LIGHT expressing tumors. However, the only tumors that experienced regressions were those treated with LIGHT and ATC or BATs. The maximum tumor response was experienced by LIGHT expressing tumors treated with BATs (Fig. 7B). In comparison, without LIGHT expression, anti-EGFR treatment was similar to BATs treatment on HT29 tumor volume (Fig. 6A). Thus, it appeared that LIGHT expression could synergize with BATs to support T-cell proliferation and impart greater tumor regressions than anti-EGFR alone.

Whether LIGHT could increase TIL was evaluated by direct tumor imaging of harvested tumors since the tumors were too small for flow cytometry processing. This revealed that the density of T-cells was markedly increased in the LIGHT expressing tumors (Fig. 7C–F). Though the tissue imaging methods were slightly different, T-cell density was calculated in the same fashion utilizing T-cell number per mm^2^ of tumor. When comparing ATC density in HT29 compared to HT29LIGHT tumors, LIGHT exposure resulted in a robust increase in ATC and BAT TILs. LIGHT increased CD4+ TIL, which BAT could not in wild-type tumors without LIGHT augmentation. The impact seen in the CD8+ TIL subset was also substantial where LIGHT significantly increased CD8+ TIL in tumors treated with ATC alone, indicating that LIGHT alone could traffic CD8+ ATC into the tumor similar to BsAb (Fig. 7E).

## Discussion

Despite improvements in colorectal cancer care, it remains the second most common cause of cancer-related death with an estimated survival of 30 months in the metastatic setting [1, 38]. CRC patient survival is accurately determined by the number and location of tumor infiltrating lymphocytes (TIL) trafficking to primary tumors [7, 10–13], and we previously demonstrated a similar association in colorectal liver metastases [16, 17]. Based on these analyses, we characterized TIL in the TME revealing that only limited numbers of ATC were infiltrating into metastases, and that tumor regressions only occurred when cytotoxic CD8 T-cells penetrated the lesions [18, 19]. When CD8+ TIL could be increased in these tumors using LIGHT (TNFSF14), durable anti-tumor regression with immune-recall responses resulted [18, 20]. Furthermore, it has been established that the patients most responsive to checkpoint blockade are those whose tumors demonstrate increased TIL [39, 40]. Though we and others have demonstrated unprecedented responses with checkpoint blockade in other cancers [41, 42], clinical trials have revealed that checkpoint blockade does not impart clinically meaningful responses in microsatellite stable (MSS, >95%) colon cancer, which is felt to have a lower neoantigen load than microsatellite instable (MSI-H) CRC [43, 44]. Thus, to improve outcomes and empower the potential of combination immunotherapy, we endeavored to examine a new strategy to increase TIL in these tumors.

The strategy that made the most sense was to physically traffic T-cells into the TME agnostic to barriers including tumor stroma, fibrosis, tumor-associated antigens, microsatellite status, or immunoediting. BsAb have been successful in recruiting TIL by simultaneously binding immune cells and cancer-specific antigens [23]. Thus one arm of the antibody would be anti-CD3. For the other arm, we turned to anti-EGFR. EGFR is naturally overexpressed in the vast majority of colon cancers, and it is already targeted in CRC with two FDA-approved antibodies, including Cetuximab. Therefore, we considered EGFR as an ideal target to draw the BsAb to the tumor, and one that could be exploited for surface binding that was not dependent on downstream signaling for efficacy. Having established that both CT26EGFR and HT29 had intact EGFR downstream signaling pathways, we expected Cetuximab monotherapy to result in anti-tumor activity, but used it as a control arm to differentiate the immune pertinent impact of BsAb treatment. Therefore, size differences in tumors were not expected between anti-EGFR treatment and BsAb treatment. Arming the BsAb with ATC would turn every ATC into a non-MHC-restricted EGFR-specific cytotoxic T-lymphocyte, create an immune microenvironment supportive of T-cell responses, contribute to in vivo BsAb viability, and avoid concerns for cytokine storm [28]. This study demonstrates our first evidence of in vivo T-cell recruitment with this antibody [33, 37].

The BATs strategy resulted in increased T-cell trafficking into murine and human colon cancer micro-island organoids and mediated increased T-cell cytotoxicity with decreased tumor cell viability in murine and human colorectal cancers. In vivo, in a syngeneic model with a competent immune system, BATs treatment enhanced T-cell trafficking into the tumors and generated anti-tumor immunity with a recall response. As a monotherapy, anti-EGFR treatment is only effective long-term in a small number of patients due to primary or secondary acquired resistance, even in patients that initially respond [45]. Therefore, as compared to anti-EGFR therapy alone, which had a similar impact on tumor volume, that BATs treatment established an anti-tumor immune response that could reject tumor rechallenge is critical to improving overall and disease-free survival [7, 46, 47].

Further evaluation in human CRC tumors revealed that BATs increased the number of CD8+ TIL, which we have previously identified as being associated with improved outcomes in this disease [13, 18, 19]. Consistent with the ability of this strategy to reject tumor rechallenge, these TIL also included increased populations of cytotoxic memory T-cells. Prior studies revealed that central memory CD8 T-cells are responsible for antigen recall and improved disease-free survival [48], however, strategies to increase memory T-cells have remained to be identified, particularly in “cold” MSS gastrointestinal tumors. Therefore, this immunotherapy approach that may increase the memory T-cell subset holds promise as exemplified in the GOLFIG-2 phase II clinical trial where increased numbers of CD8+ CCR7+ cells in the TME were associated with improved overall and disease-free survival in CRC patients [16, 18, 49–51].

Given that we had previously demonstrated anti-tumor immune responses, increased TIL, and support of TIL proliferation in CRC with tumor necrosis factor superfamily LIGHT [16, 18, 51], we endeavored to combine LIGHT and BATs together. In this first in vivo model using an inducible vector of human LIGHT in human colorectal cancer xenografts, we combined LIGHT with adoptive transfer therapy. The induction of LIGHT resulted, on average, in a 28-fold decrease in tumor growth in all treatment groups, indicating that human LIGHT has both an immune dependent and independent anti-tumor effect. Further, immunohistochemistry of treated tumors revealed a 14-fold increase in CD8+ TILs and an 84-fold increase in CD4+ TILs with ATC adoptive transfer in the LIGHT expressing tumors. While LIGHT exposure resulted in increased trafficking of TILs with both ATC and BATs, the in vitro data suggest that BsAb treatment further increased cytotoxicity of recruited TILs, and that LIGHT treatment further increases BAT proliferation. Wherein the armed BsAb strategy provided potent decreases in the rate of tumor growth and trafficked increased TIL, the addition of LIGHT imparted actual tumor regressions and increased not only CD8+ TIL, but also CD4+ TIL, which BATs alone did not. Furthermore, the addition of LIGHT to BsAb treatment enhanced T-cell proliferation above that seen when BATs were repeatedly exposed to cancer targets in vitro [52].

There are certain limitations in this study, the first being inherent to the study of human immunology in murine models. In an attempt to address this limitation, we performed our experiments in both a syngeneic immunocompetent model and in an immunodeficient xenograft model utilizing NSG mice. The anti-CD3 x anti-EGFR construct in the clinical setting is limited to EGFR expressing cancers, however, the majority of CRC tumors overexpress EGFR [21, 22]. Furthermore, downstream EGFR signaling is not necessary for BsAb function, thereby increasing the number of patients for whom this strategy would apply [22]. Finally, some anti-tumor effect observed with BATs was a result of anti-EGFR signaling, especially given the presence of intact EGFR signaling for both CT26EGFR and HT-29. Therefore, Cetuximab was used as a control in all in vivo experiments. Furthermore, tumor regressions in human CRC cells treated with BATs + LIGHT were similar to LIGHT + ATC treatment, likely due to LIGHT’s ability to increase CD8+ ATC tumor infiltration. One could hypothesize that BATs require the interplay of other immune cells not present in the immunodeficient model to support additional anti-tumor responses. Furthermore, these ATCs were already significantly activated ex vivo and supported by IL2 in the TME. Additionally, in vitro trafficking of T-cells into HT29 organoids was increased in the BAT groups, but not substantially more so after the addition of LIGHT expression despite increased proliferation and cytotoxicity with LIGHT. One could speculate that lower infiltration in this assay may be secondary to LIGHT-induced apoptosis and cytotoxicity resulting in decreased EGFR expression/availability on the cell surface of the HT29 cells in the organoids.

In conclusion, BATs promote trafficking of cytotoxic ATC into colorectal cancer tumors, and generate endogenous anti-tumor immunity and central memory T-cells in clinically relevant models. LIGHT mediates both immune dependent and independent anti-tumor function, which can be harnessed to augment ATC and BATs anti-tumor properties. One of the obvious implications of being able to successfully increase trafficking of TILs is to sensitize CRC to immune checkpoint blockade inhibitors. The innovative technique holds promise for advanced and metastatic colorectal cancer, warranting further investigation as mono or combination therapy.

## Materials and methods

### Mice, diet, and cell lines

Wild type female BALB/c mice and NSG mice were purchased from Charles River Laboratory (Wilmington, MA, USA). All experimental protocols followed NIH Guidelines and were approved by the Institutional Animal Care and Use Committees of University of Illinois at Chicago. All mice were used for experiments at age 6 to 8 weeks. According to the power analysis, where expected biologically significant differences are greater than 25% with a standard deviation of 20% utilizing an alpha of 0.05 and power of 0.8; at least six animals were placed in each experimental group. Doxycycline diet (TD.08434) was purchased from Envigo (Madison, WI, USA). CT26 murine and human HT-29 colorectal cancer cell lines were obtained from the American Type Culture Collection (ATCC, Manassas, VA, USA) and were grown in DMEM culture medium (Life technology, Grand Island, NY, USA) supplemented with 10% fetal bovine serum (Invitrogen, Waltham, MA USA). EGFR5, mouse EGFR with site mutations enabling Cetuximab binding [53] was very kindly gifted by Dr. Yang-Xin Fu. Cell lines were recently authenticated prior to in vivo use and tested for mycoplasma contamination. cDNA was cloned into XhoI sites of the pLVX-IRES-puro parental plasmid (Fig. 1A). After the pLVX-EGFR5 vector was identified through DNA sequencing, wtCT26 cells were transfected with the vector via lipofectamine 2000 and selected with puromycin (Fig. 1B). HT29 human CRC with high intrinsic expression of EGFR (Fig. 1C) was transfected with an inducible LIGHT expressing vector. The LIGHT vector was constructed by inserting the human LIGHT cDNA into BamHI/EcoRI of pLVX-TRE3G plasmid (Fig. 1A). Next, HT-29 cells were transfected with pTet3G DNA and pLVX-TRE3G-hLIGHT sequentially and selected with G418 and puromycin, respectively, to establish a HT-29hLIGHT^i^ cell clone with high expression levels of LIGHT (Fig. 1C).

### Antibodies, enzymes, reagents, and kits

Purified anti-mouse CD3 (145-2C11) and anti-mouse CD28 (37.51) mAbs were purchased from BD PharMingen (San Diego, CA). Donkey anti-human IgG PE, antiCD45 FITC, anti-CD8 Pecy7, anti-CD3 APC, anti-CD4 PE, anti-CD25 Pacific blue, anti-CD69 Percp-cy5.5, anti-CD49b Pecy7, anti-CD11c pacific blue, anti-CD80 PE, anti-CD86 APC, and Annexin V Apoptosis Detection Kit were purchased from eBiosciences (San Diego, CA, USA). Pacific blue conjugated anti-mouse CD4 (GK1.5) was purchased from Biolegend (San Diego, CA, USA). Anti-mouse LIGHT was purchased from R&D system (Minneapolis, MN, USA). Anti-mouse CD3 was purchased from Abcam (Cambridge, MA, USA) and HRP-conjugated goat anti-rabbit IgG were purchased from Santa Cruz Biotechnology (Santa Cruz, CA). CalPhos™ Mammalian Transfection Kit, Lenti-X Tet-One Inducible Expression Systems, G418, and doxycycline were purchased from Clontech (Mountain View, CA, USA). Plasmid DNA purification kit was purchased from QIAGEN (Valencia, CA) and BD Bioscience (San Jose, CA, USA.), CellTrace™ Far Red Cell Proliferation Kit, CellTrace™ CFSE Cell Proliferation Kit, Lipofectamine® 2000 Transfection Reagent, puromycin, and Trizol reagent were purchased from Invitrogen (Waltham, MA USA). Cell titer 96 aqueous non-radioactive cell proliferation assay kit and CytoTox G1780 non-radioactive cytotoxicity assay kit were purchased from Promega (Madison, WI, USA). LTβR-Ig was kindly gifted by Dr. Yang-Xin Fu. Collagenase IV and DNase I were purchased from Sigma (St. Louis, MO, USA). Restriction endonucleases enzymes including MluI, PstI, BamHI, EcoRI, T4 DNA ligase, and DH5 alpha competent E. coli cells were purchased from New England Biolabs (Ipswich, MA, USA). Phospho-EGFR reactive to EGFR/EGFR5 (Y1068) (D7A5) (Catalog number:3777 s), AKT, and ERK mAbs were purchased from Cell Signaling Technology (Danvers, MA, USA). Anti-β actin was bought from Santa Cruz Biotechnology (Dallas, TX, USA). Anti-EGFR (Cetuximab) was bought from Eli Lilly (New York, NY, USA).

### Identification of CT26EGFR and HT29hLIGHT^i^ cell lines

1 × 10^6^ of CT26EGFR cells were harvested and suspended in 100 µl PBS containing 0.5% fetal bovine serum, incubated with anti-EGFR at 1 µg/ml at 4 °C for 60 min and then incubated with anti-rabbit IgG-488 at 1:500. Cells were analyzed with a cyan ADP analyzer (Beckman Coulter, Brea, CA, USA) after multiple washes. Events were collected and analyzed using Flow Jo software (Tree star Incorporated, Ashland, OR USA) (Fig. 1B). HT29hLIGHT^i^ cells (1 × 10^6^) were harvested 24 h after incubation with or without doxycycline at 1 µg/ml and suspended in 100 µl staining buffer. Cells were incubated with anti-EGFR or incubated with LTβR-Ig at 1 µg/ml at 4 °C for 60 min, followed by incubation with anti-rabbit-488 or anti-human IgG-PE at 4 °C for 30 min. The cells were analyzed as above (Fig. 1C, D).

### Stimulation of CT26EGFR and HT-29hLIGHT^i^ cells with EGF and Western blot

CT26EGFR and HT-29hLIGHT^i^ (4 × 10^5^ cells/well) were seeded into 6 well plates on day 0, and then cells were serum starved overnight in 1% FBS RPMI 1640 medium on day 2. On day 3, the cells were stimulated with human EGF at 100 ng/ml at 37 °C for 5 and 15 min in plain RPMI medium. Cells were lysed with RIPA buffer and protein concentrations in the cell lysates were determined using a Bio-Rad protein assay kit (Bio-Rad, Hercules, CA, USA). Cell lysates of 25 µg protein were resolved on sodium dodecyl sulfate-polyacrylamide gel electrophoresis and transferred to PVDF membranes (Bio-Rad, Hercules, CA, USA). Blots were blocked for 1 h at room temperature in phosphate-buffered saline (PBS) containing 5% bovine serum albumin (BSA) and 0.1% Tween 20. Membranes were incubated 2 h with specific antibodies, washed three times in PBS containing 0.1% Tween 20, and detected using HRP-conjugated goat anti-rabbit IgG or rabbit anti-mouse IgG. After three washes in PBS containing 0.1% Tween 20, signals were revealed by an enhanced chemiluminescence detection system (Amersham) and visualized by autoradiography.

### Cross linking of anti-EGFR x anti-CD3 for murine and human BsAb generation

Mouse anti-CD3 and anti-EGFR (cetuximab) were cross-linked with the protein-protein crosslinking kit (Thermo scientific, catalog number: 23456. Rockford, IL, USA). Briefly, for the anti-CD3 maleimide activation, 5 mg purified anti-mouse CD3 (145-2C11) (ebioscience, San Diego, CA, USA) was dialyzed against PBS to thoroughly remove Tris or glycine overnight. 82.5 µl of the Sulfo-SMCC solution was added to 5.0 ml anti-CD3 solution and incubated for 30 min at room temperature. Non-reacted Sulfo-SMCC from the maleimide-anti-CD3 reaction mixture was removed through the equilibrated desalting column. For cetuximab sulfhydryl modification, 4.5 mL of polyclonal cetuximab containing 5 mg Protein (Millipore, Temecula, CA, USA) and 500 μL of Conjugation Buffer (10X) was added to the activated Immobilized Reductant and mixed thoroughly. The mixture was incubated for 20 min at 65 °C. After centrifuge, the supernatant containing sulfhydryl- cetuximab was mixed with maleimide-anti-CD3 in approximately equal molar amounts and incubated for 60 min at room temperature for crosslinking. Crosslinking of anti-EGFR x human anti-CD3 for human BsAb generation was performed using the same steps.

### ATC purification, activation, and expansion

Murine T cell activation and expansion were performed in 6 well plated coated with anti-CD3 at 2 µg/ml and soluble anti-CD28 (2 µg/ml) plus mIL-2 (100 U/ml). 5 ml of T-cells at 2 × 10^6^ cell/ml was seeded into a single well. On day 2, cells were passaged to a 10 cm dish, at 1 × 10^6^ cells/ml containing 100 U/ml murine IL-2. On day 4, 10 ml of complete T cell medium with 100 U/ml murine IL-2 was added to the dish. Cells were harvested on day 5.

Blood collection and use of human blood products for research were conducted under Institutional Review Board approved protocols at University of Virginia, and signed consents were obtained from normal and patient donors. ATC were generated in RPMI and cryopreserved. 25 ml of blood containing 1000 U/ml heparin was placed into Ficoll-Hypaque with an equal volume of 1X PBS. The interface with mononuclear cells was transferred to new 50 ml conical tube. After two PBS washes, cells were suspended in RPMI 1640. 1–1.5 × 10^6^ PBMC/mL in culture were activated with 20 ng/ml of anti-human CD3 (OKT3) and 100IU rIL-2. Cells were expanded for up to 14 days ± 3 days.

### Assessment of the dual binding activity of BsAb anti-CD3 x anti-EGFR

To identify anti-CD3 x anti-EGFR BsAb binding, different doses (25, 50, 100, 200, 500, 1000 ng) of BsAb were added to 1 × 10^6^ CT26EGFR5 cells and mouse splenocytes, respectively, and incubated at 4 °C for 1.0 h. 0.25 µl (200x dilution) anti-Armenian Hamster IgG –FITC or 0.2 µl anti-rabbit IgG-FITC (250x dilution) were added to splenocytes or CT26EGFR5 cells after washing and incubated at 4 °C for 30 min in one pot. Cells were analyzed using a cyan ADP analyzer (Beckman Coulter, Brea, CA, USA) after two subsequent washes. Events were collected and analyzed using Flow Jo software (Tree star Incorporated, Ashland, OR, USA).

To identify the human BsAb (anti-CD3 x anti-EGFR) the method and procedures are similar to the murine BsAb identification except that human T cells and human colorectal cancer cells (HT29) were utilized, and the secondary antibodies were anti-mouse-IgG-PE and anti-human-IgG-PE.

To further demonstrate that the BsAb binds simultaneously specifically to both CD3 T-cells and colon cancer cells, murine or human CD3+T cells and murine CT26EGFR5 or human HT-29 cells were labeled with cell tracker violet and far red, respectively. The labeled T cells (5 × 10^5^) and CT26EGFR5 or HT-29 cells were be mixed at 5:1 (T: CT26EGFR5 or T:HT-29) and incubated with BsAb at 1 µg/ml 1 h at 4 °C in the presence or absence of excess amounts of anti-CD3 (10 µg/ml), anti-EGFR (10 µg/ml), or both antibodies. The amount of Violet^+^Far red^+^ cell clusters were analyzed by flow cytometry.

### Arming activated T cell with BsAb

Murine CD3^+^ T cells were suspended with 2% FBS, 1xPS/AmpB, HEPES RPMI1640 at 10 × 10^6^/ml and 200 ng/ml murine BsAb, the mixture was incubated at 4 °C for 1 h and then washed twice with T-cell medium before use. Predetermined optimal arming dose of human BsAb is 50 ng/million T-cells/ml. ATC were re-suspended at 5–10^6^ T cells/ml in RPMI Medium 1640 with 10% FCS/FBS, 1% L-Glutamine and 2% penicillin/ streptomycin in 15–50 ml conical tube. BsAb and ATC were incubated for 15 min at room temperature, then washed twice with complete RPMI prior to use.

### Migration and binding of BATs to micro-island organoids

CT26EGFR cells were stained in PBS at 1 × 10^6^ cells/ml containing 1 µl/ml of far red at 37 °C for 20 min and then incubated in 5 times original staining volumes of 2% FBS RPMI 1640 for another 5 min. Pellets were re-suspended in complete medium to 0.67 × 10^6^ cells/ml. 500 µm micro-island cultures were lithographically patterned and spaced 1200 µm apart as described previously [54]. 23,000–25,000 tumor cells are organized in 90 islands within each well. On the second day, ATC, BsAb armed ATC or a mix of anti-CD3 (500 µg/1 × 10^6^ cells/100 µl) and anti-EGFR (500 µg/1 × 10^6^ cells/100 µl) armed ATC were labeled with CFSE prior to being co-cultured with CT26EGFR and HT-29hLIGHT^i^ micro-island organoids. HT29hLIGHT^i^ human colon cancer cells were cultured in the presence or absence of doxycycline at 1 µg/ml for 24 h, used as HT29LIGHT and HT29 cells, respectively, and then harvested and stained with far red after which they were seeded. The cells were fixed with 300 µl of 4% paraformaldehyde after 1.0 h incubation at 37 °C and rinsed twice with PBS. Pictures were taken under fluorescence microscope at 40x and 100x.

### Non-radioactive cytotoxicity assay

Cytotoxicity mediated by ATC or BATs to CT26 or CT26EGFR and HT-29 or HT-29LIGHT^i^ cells was performed with CytoTox G1780 Non-Radioactive Cytotoxicity assay from Promega (Madison, WI, USA). In brief, cancer cells were plated in 96 well plates, followed 4 h later by effector cells (murine or human ATC and armed ATC) at 2.5 × 10^4^, 5 × 10^4^ or 1 × 10^5^ cells/well overnight in 37 °C CO_2_ coculture. After cell lysis, supernatants were reconstituted in substrate mix and cytotoxicity signal quantified at 490 nm per manufacturer instructions.

### Proliferation assay of BsAb-armed ATC

CT26, CT26EGFR5, and HT29LIGHT^i^ (3 × 10^5^ cells/well) were seeded into 6 well plates and cultured overnight. HT29hLIGHT^i^ human colon cancer cells were cultured in the presence or absence of doxycycline at 1 µg/ml for 24 h and used as HT29LIGHT and HT29 cells, respectively. Murine or human anti-CD3 (500 ng/ml), anti-EGFR (500 ng/ml), murine or human ATC (activated T cells, 3 × 10^6^ cells/well), murine or human armed ATC (3 × 10^6^ cell/well) with anti-CD3 plus anti-EGFR, and murine or human armed ATC with BsAb (BAT) (3 × 10^6^ cells/well) were added into the wells in triplicate. Cells were harvested and counted with trypan blue exclusion 42 h after coculture. The fold change of CT26/CT26EGFR, or HT-29/HT-29LIGHT cell numbers were calculated by comparison with the original seeded cell number of CT26 or CT26EGFR and HT29 or HT29LIGHT.

### Human ATC or BATs proliferation assay in vitro

2 × 10^5^ cells ATC or BATs were co-cultured with 20,000 rad irradiated 2 × 10^4^ HT-29 or HT-29LIGHT cells in U-bottom 96 well plates for 96 h. 20 µl of MTS solution was added to each well containing 100ul of medium and absorbance was measured at 490 nm after 1–4 h incubation.

### Mouse models of CT26EGFR colorectal cancer

5 × 10^6^ CT26EGFR cells in 100 µL PBS were inoculated subcutaneously in the right flank of Balb/C mice. When the tumor size reached ~150 mm^3^, animals were randomized into for equal numbers of animals in each group, and each animal was adoptively transferred in a non-blinded fashion with one of four treatments: 100 µl of RPMI, ATC or BATs with 3000U murine IL2, via retro orbital vein injection or anti-EGFR via intraperitoneal injection for four dosages with 7 day intervals. Tumor volumes were calculated using the equation ½ (long diameter × short diameter [2]). One part of the tumor was fixed in 10% formalin for immunohistochemical staining and another part of the tumor was dissociated with collagenase IV. The acquired single cell suspension (1 × 10^6^/sample) was stained with conjugated antibodies and analyzed with FACS. The tumor infiltrating lymphocytes were analyzed after tumor tissues were stained with murine anti-CD3 followed by conjugated goat ant-rabbit IgG immunofluorescence staining using Image J.

For the CT26EGFR rechallenge experiment, 14 days after last treatment, 5 × 10^6^ CT26EGFR cells in 100 µL PBS were inoculated subcutaneously in the left flank of the mouse. The mice were sacrificed on day 13 after rechallenge.

### Mouse models of HT-29hLIGHT^i^ colorectal cancer

6-week-old NSG mice were randomly divided into 8 groups of at least 6 mice per group. 5 × 10^6^ HT-29hLIGHT^i^ cells in 100 µL PBS were inoculated subcutaneously in the right flank of NSG mice. When the tumor size reached to ~150 mm^3^, half of the animals were randomized to have LIGHT expression induced, as previously [18]. Treatments were now not blinded. Groups 1 and 5 were injected with 100 µl of RPMI containing 3000U hIL2 per mouse via retro orbital vein. Groups 2 and 6 were injected with 200 µg anti-EGFR intraperitoneally. Groups 3 and 7 received 20 × 10^6^ human activated T cells in 100 µl RPMI containing 3000U hIL-2 via retro orbital vein. Groups 4 and 8 received 20 × 10^6^ of BATs in 100 ul RPMI containing 3000 U hIL-2 via retro orbital vein. Each mouse was given 1 cycle of treatment every 7 days for 6 cycles. Mice were sacrificed 48 h after last treatment, and analysis was as in murine tumors.

### Immunohistochemistry

Tumor tissues were collected at the indicated times, fixed in 10% neutral buffered formalin, embedded in paraffin, and stained with hematoxylin and eosin (H&E) and murine anti-CD3 antibody (1:750) (Ab11169, Abcam, and Cambridge, MA, USA) per protocol. Photographs were obtained with an Olympus BX51 fluorescence microscope and images were acquired using an Olympus DP71 CCD camera and DP Controller Software (Olympus, Center Valley, PA, USA). Whole tumor quantification was performed with inForm® software and confirmed with manual blinded quantification according to our standard clinical pathology protocol for primary colon cancers. In brief, the tumor was reviewed at low power and an area with the most TIL or lymphocytes was identified. In this location, 10 consecutive 400X images were evaluated and only cells infiltrating between tumor cells were counted. They were counted in a blind fashion with the tumor margin considered a maximum width of 360 µm from the edge of the tumor [55].

Xenografts underwent antigen retrieval and then slides were subjected to sequential staining with CD8 antibody (RTU, clone C8/144B, Biocare Medical, Pacheco, CA), CD3 (1:100, clone SP7, Abcam, Cambridge, UK), and CD4 (1:100, clone EP204, Sigma Aldrich, St. Louis, MO). Blocking, detection, and DAPI staining were performed with reagents from OpalTM 4-color Automation IHC Kit (Akoya Biosciences, #NEL820001KT, Menlo Park, CA). A spectral library was prepared using human tonsil control slides and pan-CK antibody (Clone AE1/AE3, #M3515, Agilent, Santa Clara, CA). Spectra of all library components were obtained using Vectra 3.0 Automated Quantitative Pathology System (Akoya Biosciences). Stained samples were scanned at 20X on Vectra 3.0 instrument and the spectral library was used to spectrally unmix images in inForm® software (Akoya Biosciences) for visualization of each color and to assess quality of each image. Unmixed images were used to segment cells and train inForm® software to phenotype and count CD3+/CD8+ and CD3+/CD4+ T-cell populations. The data were exported and analyzed in R where T-cell population densities (number of positive cell/mm^2^) were calculated.

### 3-dimensional multiplex confocal microscopy

Conjugation of fluorescent dyes to primary antibodies and tumor tissue preparation for imaging was as previously described [56]. Briefly, frozen tumor tissues in optical cutting temperature (OCT) compound were thawed at room temperature. Samples were embedded in 2% agarose and sectioned with a vibrating microtome (600 µm thickness). The following primary antibodies were conjugated to specific DyLight (DyL) dyes (Thermo Fisher): anti-human EGFR (528 clone, BioXCell), anti-human CD8 (HIT8a clone, Thermo Fisher), anti-human CD4 (RPA-T4 clone, Biolegend), anti-human CD3 (HIT3a clone, Biolegend), and anti-human CD31 (WM59 clone, Biolegend). Individual tissue sections were stained overnight with one of two panels: Panel 1) DAPI, DyL488-anti-hEGFR, DyL550-anti-hCD8, and DyL680-anti-hCD3; Panel 2) DAPI, DyL488-anti-hEGFR, DyL550-anti-hCD4, and DyL680-anti-hCD3. Sections were made optically transparent by room temperature incubation in 20% and 80% fructose solutions. All samples were imaged with a Caliber ID RS-G4 upright confocal microscope under the same laser settings: 405 nm excitation and 450/70 nm emission filter for DAPI; 488 nm excitation and 520/44 nm emission filter for DyL488-hEGFR; 561 nm excitation and 600/52 nm emission filter for DyL550-hCD8 and DyL550-hCD4; 640 nm excitation and 700/75 m nm emission filter for DyL680-hCD3. Multiple high-resolution fields of view were captured throughout each section using a Zeiss 40X water immersion objective (NA1.1; WD510 µm). We implemented FIJI ImageJ (imagej.net/Fiji) functions to threshold fluorescence intensity, pinpoint double positive CD3+ CD8+ or CD3+ CD4+ human T cells and calculate cell counts. Processed images were manually checked for accurate counting. In order to obtain representative 3D tumor images, we stained tumor microsections with DAPI, DyL488-anti-hEGFR, DyL550-anti-hCD8, DyL594-anti-hCD3, and DyL633-anti-hCD31, and imaged using a Leica TCS SP8 confocal microscope with the following laser settings: 405 nm excitation and 420–455 nm emission filter for DAPI; 488 nm excitation and 495–530 nm emission filter for DyL488-hEGFR, 550 nm and 563–579 nm filter for DyL550-hCD8, 594 nm and 603–624 nm filter for DyL594-hCD3, 633 nm and 648–662 nm filter for DyL633-hCD31. We constructed 3D visualizations of the tumor microsection images using Imaris software (Bitplane).

### Statistical analysis

All microscopic images and manual counts were performed in a blind fashion. Statistical analysis was performed on data that met the assumption of the test and with similar variance between groups that are being compared, with two tailed Student’s t-test. Error bars represent SEM. *P < 0.05, **p < 0.01, and ***p < 0.001. Statistical significance was considered for *p*-value < 0.05. Criteria to include samples with biologic variance within two standard deviation of the mean were pre-established. All statistical analysis was performed with Microsoft Excel**®**.

## Data Availability

The authors are committed to making data and material publicly available to the scientific community.
